# Implementing a bedside assessment of respiratory mechanics in patients with acute respiratory distress syndrome

**DOI:** 10.1186/s13054-017-1671-8

**Published:** 2017-04-04

**Authors:** Lu Chen, Guang-Qiang Chen, Kevin Shore, Orest Shklar, Concetta Martins, Brian Devenyi, Paul Lindsay, Heather McPhail, Ashley Lanys, Ibrahim Soliman, Mazin Tuma, Michael Kim, Kerri Porretta, Pamela Greco, Hilary Every, Chris Hayes, Andrew Baker, Jan O. Friedrich, Laurent Brochard

**Affiliations:** 1 Interdepartmental Division of Critical Care Medicine, University of Toronto, Toronto, ON Canada; 2 Keenan Research Centre and Li Ka Shing Institute, Department of Critical Care, St. Michael’s Hospital, 209 Victoria Street, Room 408, Toronto, ON M5B 1T8 Canada; 3 Department of Critical Care Medicine, Beijing Tiantan Hospital, Capital Medical University, Beijing, China; 4 Department of Respiratory Therapy, St. Michael’s Hospital, Toronto, ON Canada

**Keywords:** Pulmonary function test, Respiratory physiology, Esophageal pressure, Mechanical ventilation, Quality improvement

## Abstract

**Background:**

Despite their potential interest for clinical management, measurements of respiratory mechanics in patients with acute respiratory distress syndrome (ARDS) are seldom performed in routine practice. We introduced a systematic assessment of respiratory mechanics in our clinical practice. After the first year of clinical use, we retrospectively assessed whether these measurements had any influence on clinical management and physiological parameters associated with clinical outcomes by comparing their value before and after performing the test.

**Methods:**

The respiratory mechanics assessment constituted a set of bedside measurements to determine passive lung and chest wall mechanics, response to positive end-expiratory pressure, and alveolar derecruitment. It was obtained early after ARDS diagnosis. The results were provided to the clinical team to be used at their own discretion. We compared ventilator settings and physiological variables before and after the test. The physiological endpoints were oxygenation index, dead space, and plateau and driving pressures.

**Results:**

Sixty-one consecutive patients with ARDS were enrolled. Esophageal pressure was measured in 53 patients (86.9%). In 41 patients (67.2%), ventilator settings were changed after the measurements, often by reducing positive end-expiratory pressure or by switching pressure-targeted mode to volume-targeted mode. Following changes, the oxygenation index, airway plateau, and driving pressures were significantly improved, whereas the dead-space fraction remained unchanged. The oxygenation index continued to improve in the next 48 h.

**Conclusions:**

Implementing a systematic respiratory mechanics test leads to frequent individual adaptations of ventilator settings and allows improvement in oxygenation indexes and reduction of the risk of overdistention at the same time.

**Trial registration:**

The present study involves data from our ongoing registry for respiratory mechanics (ClinicalTrials.gov identifier: NCT02623192. Registered 30 July 2015).

**Electronic supplementary material:**

The online version of this article (doi:10.1186/s13054-017-1671-8) contains supplementary material, which is available to authorized users.

## Background

Patients with acute respiratory distress syndrome (ARDS) present various degrees of impairment in respiratory mechanics and different physiological responses to a given level of positive end-expiratory pressure (PEEP). Applying the same ventilator regimen to every patient would be inadequate and at times can be potentially harmful. For example, the potential benefits of high PEEP in terms of oxygenation improvement and alveolar recruitment should be balanced against the risks induced by high pressures, such as hemodynamic impairment and overdistention. In other words, one needs to individualize the PEEP level by evaluating both its safety and its effectiveness for a specific patient [1]. This requires the assessment of gas exchange, respiratory mechanics, and hemodynamic variables. Additionally, partitioning lung and chest wall mechanics can also help the individualization of ventilator settings.

Despite their potential interest for clinical management, neither airway pressure (Paw)-based respiratory mechanics nor esophageal pressure (Pes)-based lung and chest wall mechanics are systematically assessed in routine practice. This discrepancy can be explained by technical issues [2] in obtaining accurate transpulmonary pressure (P_L_), by a lack of standardized procedures, and by the challenges of integrating the results of these measurements into ventilatory management. Even worse, the most recent large observational studies on patients with ARDS showed that simple parameters such as plateau pressure (Pplat) were not measured in the majority of the patients [3].

To improve the integration of respiratory mechanics measurement in our clinical practice, a group of physicians and respiratory therapists (RTs) at our institution introduced a respiratory mechanics test to systematically assess respiratory mechanics (i.e., performing a pulmonary function test) for patients with ARDS and be implemented as a quality improvement (QI) program. The goal was to provide clinicians with relevant physiological assessment that could be helpful for clinical practice. Because the needs for adjusting ventilator settings can be very different, this program did not include clinical recommendations or specific guidelines associated with these measurements.

Having implemented this systematic test in our clinical practice for 1 year, we retrospectively tried to assess if it had any impact. We looked for whether any changes in ventilatory settings were performed. We also assessed whether the observed changes modified physiological variables known to be associated with mortality, and we tried to understand whether the observed changes were consistent with the measurements.

## Methods

### Design and settings

This is a retrospective study of the impact of a 1-year program (see below) with an aim of systematically evaluating respiratory mechanics in patients with ARDS by comparing the ventilator settings and relevant physiological variables before and after performing the measurements. The program was decided by the critical care department at a teaching hospital (St. Michael’s Hospital, Toronto, ON, Canada) and implemented in both the medical-surgical and the trauma-neurosurgical intensive care units (ICUs). Of note, the measurements are entered into a registry for future studies.

### Implementing the respiratory mechanics test in clinical practice

The procedure of the test was determined through discussion among ICU physicians, fellows, and RTs. A team of 22 users (3 physicians, 2 fellows, and 17 RTs) was recruited voluntarily to facilitate implementing the test in four aspects:

- To increase awareness and understanding of monitoring respiratory mechanics, education sessions consisting of lectures, bench and bedside hands-on sessions, and feedback rounds were provided to ICU clinicians. The education sessions were focused on explaining the importance of measuring respiratory mechanics, the technical approaches for measurements, the physiological and clinical meanings of the measured variables based on scientific evidence, and the limitations of those variables. We did not propose to use one single parameter on which to base changes of the ventilator settings; we proposed to incorporate multiple variables (e.g., airway pressure, Pplat, driving pressure [Pdriv], chest wall component, recruitability, oxygenation, and hemodynamic response to PEEP) into the global history of the patient and let the clinical team decide what was best for the patient.
- To standardize the procedures, we developed written protocols to guide esophageal catheter placement and the associated systematic measurements.
- To simplify the calculations, we developed a custom-programmed Portable Document Format form (PDF; Adobe Systems, San Jose, CA, USA) to automatically calculate physiological parameters and generate a clinical report (see Additional file 1: Appendixes S1 and S2).
- This clinical report was delivered to the caregivers in charge of the patients.

### Patient enrollment process for the test

All patients admitted to the ICUs meeting the Berlin definition of ARDS [4] and receiving invasive mechanical ventilation were eligible. A daily screening was done, mostly on the weekdays. It was left at the discretion of the clinical team to decide to perform measurements, place esophageal catheters, and accept possible transient changes in sedation or paralysis (Additional file 1: Figure S1). Esophageal catheter insertion was recommended when the ratio of partial pressure of arterial oxygen and fraction of inspired oxygen (PaO_2_/FiO_2_) was ≤200 mmHg. In the group of patients with mild ARDS (i.e., PaO_2_/FiO_2_ > 200 mmHg), catheters were placed at the discretion of the clinical team. In the following cases, the clinical team discussed the benefits of doing the measurements on a case-by-case basis: (a) severe hemodynamic instability (i.e., >30% increase in the dose of vasopressors in the last 6 h or need for >0.5 μg/kg/minute of norepinephrine); or (b) a known esophageal problem, active upper gastrointestinal bleeding, or any other contraindication to the insertion of a gastric tube.

### Measurements

**Fig. 1:**
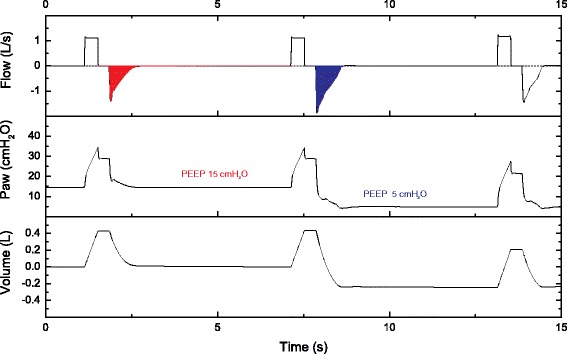
Illustration of simplified decremental positive end-expiratory pressure (PEEP) maneuver for estimating derecruited lung volume. In this example, respiratory frequency was transiently reduced to 10 breaths per minute to allow a prolonged expiration. Afterward, PEEP was reduced from 15 to 5 cmH_2_O. The difference in expiratory tidal volumes (i.e., integral of flow) between the breath while decreasing PEEP (*blue area*) and the one before changing PEEP (*red area*) was referred to as the total change in lung volume. Derecruited volume was the difference between the total changes in measured vs. predicted lung volumes (see text for details). *Paw* Airway pressure

Each patient enrolled underwent measurements of respiratory mechanics performed by one or two trained RTs and/or fellows (depending upon availability of clinicians) following a standardized protocol. More than 20 clinicians were considered as trained users. The patients were measured at the early stage of ARDS, and all of them were already deeply sedated and often paralyzed. Additional sedation with or without paralysis could be transiently necessary to suppress or minimize spontaneous breathing. This approach was accepted as part of our clinical practice to get reliable measurements of passive respiratory mechanics. Nevertheless, the decisions of deepening sedation and/or using paralysis for an individual patient were made at the discretion of the clinical team. The absence of spontaneous effort was confirmed by the absence of a negative Paw swing during a 3-second end-expiratory occlusion and by the presence of positive Pes swings during tidal breathing. Volume-controlled ventilation (VCV) was used during the measurements with a standardized tidal volume (V_T_) of 6 ml/kg of predicted body weight (PBW), a constant inspiratory flow of 50–60 L/minute, and 0.3-second pause at the end of inspiration. Respiratory rate (RR) was set to maintain a minute ventilation (V_E_) similar to premeasurement level. PEEP and the fraction of inspired oxygen (FiO_2_) were maintained at the clinically chosen level. Paw, airway flow, and airway volume were directly taken from the ventilator monitoring system. Pes was measured using a catheter with an air-filled balloon (CooperSurgical, Trumbull, CT, USA) with a pressure transducer connected to a bedside monitor (similarly to measuring central venous pressure). Measuring Pes was recommended as a component of the test for patients with a PaO_2_/FiO_2_ ratio ≤200 mmHg, but the decision of placing a catheter was left at the discretion of the clinicians. The validity of the Pes was confirmed using an occlusion test during spontaneous breathing (premeasurement) or a positive pressure occlusion test by manually compressing the thorax during passive breathing [2, 5]. Elastance, resistance, and other derived variables were automatically calculated using the programmed PDF form. The process of conducting the measurements was as follows:

- Paw-based respiratory mechanics were measured by using end-expiratory and end-inspiratory occlusions for 1–2 seconds. The absence of leakage during an end-inspiratory occlusion was confirmed by the equivalence of expiratory V_T_ between the breaths with occlusion to the one without occlusion. Total positive end-expiratory pressure (PEEPtot), airway peak pressure (Ppeak), and airway Pplat were recorded. Intrinsic PEEP, Pdriv (Pplat − PEEPtot), respiratory system compliance, and resistance were then calculated automatically.
- Pes-based lung and chest wall mechanics [2] were measured simultaneously using end-expiratory and end-inspiratory occlusions. Transpulmonary pressure at end expiration (P_L,end-exp_) and transpulmonary pressure at end inspiration (P_L,end-insp_), lung compliance, chest wall compliance, and the ratio of lung elastance to respiratory system elastance were calculated automatically. P_L_, unless specifically indicated such as elastance-derived transpulmonary plateau pressure, was calculated using direct measurement of Pes.
- Oxygenation and hemodynamic responses to PEEP were assessed by increasing PEEP by 3–5 cmH_2_O (preferably 5 cmH_2_O) from the clinical PEEP level if the Pplat was <35 cmH_2_O (in the vast majority of the cases). PEEP was reduced by 3–5 cmH_2_O if the Pplat had reached 35 cmH_2_O or in the presence of poor hemodynamic tolerance. We report this procedure as an incremental PEEP trial. FiO_2_ was kept constant for comparing the change in PaO_2_/FiO_2_.
- Alveolar derecruitment was estimated using a single-breath simplified decremental PEEP maneuver (Fig. 1) performed within 10–15 seconds from the high PEEP used in the preceding step. A prolonged expiration (6–9 seconds) maneuver was performed while abruptly decreasing PEEP by 10 cmH_2_O from a high to a low level for one breath. Because inspiratory V_T_ was unchanged, the difference in expiratory V_T_ values between the expired V_T_ displayed immediately after decreasing PEEP and the breath before changing PEEP was referred to as the total change in lung volume from high to low PEEP. In parallel, the predicted change in lung volume was estimated by the product of respiratory system compliance at low PEEP (or zero PEEP) and the change in pressure (i.e., 10 cmH_2_O of change in PEEP). When the total change in lung volume was greater than this predicted value, the difference was taken as an estimate of derecruited lung volume (V_der_). A high V_der_ (e.g., ≥150 ml) due to reducing PEEP suggested that the PEEP was effective in recruiting the lung (or in maintaining the lung recruited). The rationale for this approach was reported previously, although the current method was simplified to make its use feasible rapidly at the bedside [6]. Of note, we reduced PEEP to estimate derecruitment instead of raising PEEP to estimate recruitment because we speculated that derecruitment may occur faster than recruitment and is easier to detect. These technical simplifications have not been fully validated, however, and this was made clear to the clinicians.

A clinical report (Additional file 1: Appendix S2) was then generated automatically with reference thresholds from the literature for diagnostic purposes. At the end of the measurements, initial ventilator settings were resumed, and the clinical report was given to the clinical team in charge of the patient. The clinicians then decided whether to change the ventilator settings if deemed necessary. There were no therapeutic recommendations attached to the report, and the clinical team was free to use and integrate the data into a more cohesive clinical decision-making process. The intention of this procedure was to provide intensivists with reliable information on the pulmonary function of patients with ARDS and possibly to allow clinicians to individualize ventilator settings.

### Patient enrollment for the present analysis

Patients who had been enrolled in the program during its first year of implementation were eligible for the present study. Patients whose results of the respiratory mechanics were not provided to the clinical team after the measurements owing to technical problems (i.e., no clinical report was generated for reference) were excluded from the study.

### Endpoints for the analysis

We designed a statistical plan to decide a priori which variables we wanted to test. The first question was to determine the proportion of patients in whom ventilatory settings were changed after the measurements. We also wanted to determine if the changes were consistent with the measurements. The relevant endpoints were the effects of these changes on physiological parameters known to be associated with mortality, namely the oxygenation index (OI = mean Paw × FiO_2_ × 100/PaO_2_) [7, 8], the estimated physiological dead-space fraction [9], Pplat, and Pdriv [10]. The OI integrates the intensity of ventilator assistance in terms of delivered mean Paw and its arterial oxygenation output. A low OI means that a relatively small intensity of assistance is needed for each millimeter of mercury of PaO_2_, whereas a high OI means that high delivered pressure and/or FiO2  is needed for each millimeter of mercury of PaO_2_. The dead-space fraction was calculated using the Enghoff modification of the Bohr equation with an estimation of resting energy expenditure using the Harris-Benedict equation and an assumption that the respiratory quotient equals 0.8. It is noteworthy that these two parameters, OI and dead-space fraction, were not directly given to clinicians. The Pdriv, calculated as the difference in Paw between a 1- to 2-second end-inspiratory occlusion (i.e., Pplat) and an end-expiratory occlusion (i.e., PEEPtot), was referred to as Pdriv in this study. For the purpose of our retrospective study, however, we found that the PEEPtot was not always assessed and documented by clinicians before or after the measurements and that the Pplat was sometimes estimated from the Ppeak in patients receiving pressure-controlled ventilation (PCV). The estimated Pdriv before and after the measurements could therefore slightly differ from the Pdriv during the measurements, and it was calculated as the difference between estimated Pplat and PEEP (not PEEPtot) and referred to as *∆P* [10].

We also calculated corrected V_E_ (i.e., V_E_ for a PaCO_2_ of 40 mmHg) as a surrogate of dead space [4]. Inspired by the OI, we proposed a new index—the “Oxygenation/Stretch Index” (O/SI). Comparing it with the OI, we replaced the mean Paw with the Pdriv and switched the denominator with the nominator for easier calculation. Using Pdriv instead of mean Paw makes the index less dependent on the type of ventilator mode used (VCV vs. PCV). It was calculated using the following equation:

$$ \mathrm{O}/\mathrm{S}\mathrm{I} = \frac{\mathrm{Pa}{\mathrm{O}}_2/\mathrm{Fi}{\mathrm{O}}_2}{\Delta \mathrm{P}}\kern0.5em =\kern0.5em \frac{\mathrm{Pa}{\mathrm{O}}_2}{\mathrm{Fi}{\mathrm{O}}_2 \times \Delta \mathrm{P}} $$

The denominator can be referred to as the “cost” of mechanical ventilation, whereas the numerator is the “benefit.” Therefore, a low O/SI suggests a low “benefit-to-cost ratio” of mechanical ventilation; that is, the benefit was achieved by paying a relatively high cost, whereas a high O/SI suggests a high benefit-to-cost ratio. Both the corrected V_E_ and the O/SI before and after the measurements were compared. The O/SI was not provided to clinicians but was calculated for the study.

### Data collection and statistical analysis

We collected information by reviewing the clinical charts for patients’ demographic, physiological, and radiographic characteristics; ARDS risk factors; coexisting conditions; ventilator settings; arterial blood gas (ABG) analysis; and documented respiratory parameters (e.g., V_E_ and Pplat) before and after measurements (i.e., premeasurement and postmeasurement). We used the ABGs that were closest to the time of measurements but at least 1 h away from the measurements, as well as the corresponding ventilator settings and respiratory parameters. (Also refer to the discussion.) Detailed physiological variables obtained during the measurements were also collected.

The details of statistical analysis are reported in the additional files. Statistical methods are also described in the notes of the tables. Notably, because we decided the variables to be tested a priori in the statistical plan, we decided against using a Bonferroni adjustment, which would have highly increased the risk of type II errors [11].

## Results

During the first year of implementation (August 2014 to August 2015), 62 patients were enrolled and had measurements performed (Additional file 1: Figure S1). One patient was excluded from the study because of obvious input errors in the measurements (data were not used by clinicians).

The study cohort consisted of 61 patients. Their main characteristics are described in Table 1. Fifty-four patients (88.5%) were measured within 48 h of ARDS identification, and the remainder were measured between 48 and 120 h after ARDS identification. The main reason for a delay between identifying ARDS and performing measurements was hemodynamic instability. Paw-based respiratory mechanics were measured in all patients, and an esophageal catheter was placed in 54 patients (88.5%). In one patient, the catheter failed to pass through the upper esophageal sphincter, and in another, the catheter was electively placed with the assistance of gastroscopy. Pes was measured in 53 (86.9%) of 61 patients. A positive occlusion test was performed in all patients, with a ratio of changes in Pes and Paw during end-expiratory occlusion (∆Pes/∆Paw) of 1.1 ± 0.2 (mean ± SD). Seven patients (11.5%) were measured without obtaining ABGs for assessment of oxygenation response, and three patients (5.0%) were measured without performing a decremental PEEP maneuver for estimation of alveolar derecruitment. During the measurements, 21 patients (34.4%) received additional sedatives, and 3 patients (5.0%) required additional neuromuscular blockers to suppress or minimize spontaneous breathing. The 30-day mortality, ICU mortality, and hospital mortality rates in the study cohort were 37.7%, 37.7%, and 41.0%, respectively. ICU and hospital lengths of stay were 13.5 [8.8–29.0] and 19.0 [10.0–40.0] days, respectively.

**Table 1:** Characteristics of the patients (*N* = 61)

Characteristic	Value
Male sex, *n* (%)	48 (78.7)
Age, years	56 [45–68]
Predicted body weight^a^, kg	68 ± 11
Body mass index, kg/m^2^	29 ± 7
APACHE II score at admission^b^	28 ± 10
SOFA score at inclusion^c^	12 ± 4
Days of NIV prior to intubation, *n*	0 [0–1]
Days of IMV at inclusion^d^, *n*	2 [1–6]
Days of ARDS at inclusion^d^, *n*	0 [0–1]
Risk factors of ARDS^e^, *n* (%)	
Pneumonia	32 (52.5)
Extrapulmonary sepsis	9 (14.8)
Trauma	9 (14.8)
Noncardiogenic shock	7 (11.5)
Pancreatitis	5 (8.2)
Aspiration	4 (6.6)
Pulmonary contusion	3 (4.9)
Pulmonary vasculitis	2 (3.3)
Drug overdose	2 (3.3)
Blood transfusion	1 (1.6)
No risk factor	4 (6.6)
Severity of ARDS, *n* (%)	
Mild	9 (14.8)
Moderate	39 (63.9)
Severe	13 (21.3)
Patients treated with ECMO	3 (4.9)
Patients with tracheostomy	9 (14.8)
Days of IMV after inclusion, *n*	9 [4–20]
Duration of IMV, days	12 [7–24]

*Abbreviations: APACHE* Acute Physiology and Chronic Health Evaluation, *ARDS* Acute respiratory distress syndrome, *ECMO* Extracorporeal membrane oxygenation, *IMV* Invasive mechanical ventilation, *NIV* Noninvasive ventilation, *SOFA* Sepsis-related Organ Failure Assessment

Dichotomous or nominal categorical variables are described in number (percent); continuous variables are described as mean ± SD or median [IQR], as appropriate

^a^Predicted body weight of male patients was calculated as 50 + 0.91 × (centimeters of height − 152.4) and that of female patients as 45.5 + 0.91 × (centimeters of height − 152.4)

^b^APACHE II score at intensive care unit admission

^c^SOFA score at day of patient assessment (i.e., the day the patient was measured)

^d^The days receiving IMV before inclusion and the days of ARDS identification before inclusion, respectively

^e^Patients can have more than one risk factor. The sum of the percent is hence >100%

### Ventilator settings

**Fig. 2:**
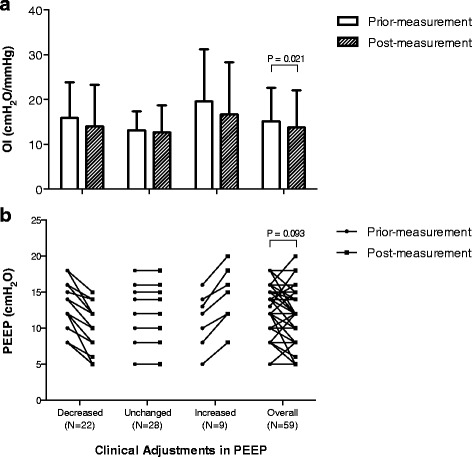
Panel **a** shows the clinical adjustments in positive end-expiratory pressure (PEEP) and effects on the oxygenation index (OI) (*n* = 59). **b** Patients were classified in three groups according to the change in their PEEP level. Bonferroni adjustment was not used. (Refer to main text for explanations.)

Comparing postmeasurement with premeasurement, the ventilator settings were found to be altered in 41 patients (67.2%) and unchanged in the others. Changes included switching pressure-targeted mode to volume-targeted mode in 19 patients (31.1%), reducing peak inspiratory pressure in 4 patients who were receiving pressure-targeted mode, decreasing PEEP in 22 patients, and increasing PEEP in 9 patients (Fig. 2). V_T_ was slightly lower in postmeasurement than in premeasurement (Table 2).

**Table 2:** Ventilator settings and physiological variables before and after measurements (*N* = 61)

	Premeasurement	Postmeasurement	*P* value
Ventilator settings			
V_T_/PBW, ml/kg	6.5 [6.2–7.0]	6.4 [6.2–6.7]	0.006
PEEP, cmH_2_O	12 [10–14]	12 [10–14]	0.077
FiO_2_	0.60 [0.50–0.70]	0.60 [0.50–0.70]	0.325
Physiological variables			
PaCO_2_, mmHg	41 [38–50]	42 [38–50]	0.553
PaO_2_/FiO_2_, mmHg	146 ± 60	162 ± 69	0.020
P_plat_^a^, cmH_2_O	30 ± 5	28 ± 5	0.004
∆P^a^, cmH_2_O	18 [14–20]	15 [12–19]	0.023
OI^b^, cmH_2_O/mmHg	15.2 ± 7.4	13.8 ± 8.3	0.021
O/SI^a^, mmHg/cmH_2_O	7.5 [5.4–11.5]	8.2 [5.9–14.7]	0.029
V_D_/V_T_, _est_	0.63 ± 0.10	0.62 ± 0.12	0.494
V_E, corr_, L/minute	13.0 ± 3.2	12.8 ± 3.3	0.421

*Abbreviations: Vt/PBW* Tidal volume per predicted body weight, *PEEP* Positive end-expiratory pressure, *FiO*_*2*_ Fraction of inspired oxygen, *PaCO*_*2*_ Partial pressure of carbon dioxide, *PaO*_*2*_ */FiO*_*2*_ Ratio of partial pressure of arterial oxygen to fraction of inspired oxygen, *Pplat* Plateau pressure, *∆P* Driving pressure, *OI* Oxygenation index, O/SI Oxygenation/Stretch Index, indicating a benefit-to-cost ratio, *V*_*D*_ */V*_*T*_ *,*_*esti*_ Estimated physiological dead-space fraction, *V*_*E,corr*_ Corrected expired volume per minute

Continuous variables were described as mean ± SD and compared using paired *t* tests or described as median [IQR] and compared using Wilcoxon matched-pairs signed-rank sum tests, as appropriate. Bonferroni adjustment was not used. (Refer to main text for explanations.)

^a^ *N* = 45; 6 patients who were ventilated in volume control mode without documented Pplat were excluded. ∆P was calculated as the difference between Pplat and PEEP

^b^ *N* = 59; 2 patients who were ventilated in pressure support mode either before or after the measurements were excluded from analysis

### Physiological outcomes

Comparing postmeasurement with premeasurement values (Table 2), both OI and distending pressures (i.e., Pplat and ∆P) were significantly reduced. Measured at the same level of FiO_2_, the PaO_2_/FiO_2_ and the O/SI were significantly increased. In patients in whom PEEP was increased, the PaO_2_/FiO_2_ increased from 124 ± 78 mmHg to 173 ± 89 mmHg (*P* = 0.010), whereas the mean Paw increased from 18 ± 3 cmH_2_O to 21 ± 3 cmH_2_O (*P* < 0.0005). In patients whose PEEP was decreased, the PaO_2_/FiO_2_ remained stable (150 ± 64 mmHg vs. 153 ± 70 mmHg; *P* = 0.772), whereas the mean Paw decreased from 20 ± 4 cmH_2_O to 16 ± 4 cmH_2_O (*P* < 0.0005) (Fig. 2). On average, for the whole group, PaO_2_/FiO_2_ improved, whereas mean Paw decreased.

The estimated physiological dead-space fraction and corrected V_E_ remained unchanged (Table 2). Arterial pH, bicarbonate level, heart rate, and mean arterial pressure remained unchanged (Additional file 1: Table S3).

### Changes in ventilator settings associated with measurements

To determine whether the observed clinical adjustments in PEEP were consistent with the results of the respiratory mechanics tests, we separated patients into three groups according to whether the PEEP was decreased by the clinicians, unchanged, or increased postmeasurement compared with premeasurement. Comparisons of the physiological variables between groups are shown in Table 3. The three groups significantly differed on four parameters, with gradually increasing values suggesting that these values could be the reason for making the changes. The airway Pplat and the elastance-derived transpulmonary Pplat were higher in the PEEP-decreased group than in the group with unchanged PEEP. P_L,end-exp_ was not different between groups. The changes in Pdriv and lung Pdriv during the incremental PEEP trial demonstrated a consistent trend among the three groups, and the lung Pdriv displayed a statistically significant difference. V_der_ was lower in the group with decreased PEEP than in the group with increased PEEP. A Spearman’s correlation analysis also confirmed that Pplat, elastance-derived transpulmonary Pplat, and the changes in lung Pdriv were correlated with the differences in PEEP before and after measurements, again suggesting that the amount and the direction of the PEEP change could be explained by values of respiratory mechanics (Additional file 1: Table S1).

**Table 3:** Relationship between measurements and subsequent clinical adjustments of PEEP

	PEEP at postmeasurement vs. at premeasurement			
Measured variables	Decreased (*n* = 20)	Unchanged (*n* = 27)	Increased (*n* = 8)	*P* value
Markers of overdistention^a^				
P_plat_, cmH_2_O	28 ± 5^b^	25 ± 4^b^	26 ± 2	0.013
Elastance-derived P_L,plat_, cmH_2_O	21 [20–26]^b^	17 [16–20]^b^	18 [16–21]	0.034
P_L,end-insp_, cmH_2_O	5 [3–18]	8 [4–9]	4 [0–10]	0.327
P_driv_, cmH_2_O	15 ± 5	12 ± 4	13 ± 3	0.098
P_L,driv_, cmH_2_O	12 ± 5	9 ± 4	9 ± 5	0.063
Risk of atelectasis^a^				
P_L,end-exp_, cmH_2_O	−2 ± 5	−2 ± 5	−5 ± 5	0.335
Response to the incremental PEEP trial^c^				
Changes in P_driv_, cmH_2_O	2.0 [0–3.5]	1.0 [0–1.0]	0.5 [0–12]	0.169
Changes in P_L,driv_, cmH_2_O	1.9 ± 2.5	0.7 ± 1.8	−0.3 ± 1.2	0.042
Changes in PaO_2_/FiO_2_, mmHg	−4 [−17 to 12]	0 [−18 to 14]	3 [−29 to 10]	0.226
Changes in MAP, mmHg	−2 [−10 to 3]	−2 [2-9]	−3 [−8 to 0]	0.675
Recruitability^d^				
V_der_, ml	105 ± 61^e^	142 ± 106	208 ± 124^e^	0.036

*Abbreviations*: *P*_*plat*_ Airway plateau pressure, *Elastance-derived P*_*L,plat*_ Elastance-derived transpulmonary plateau pressure, calculated using airway plateau pressure times the ratio of lung elastance to respiratory system elastance, *P*_*L,end-insp*_ Transpulmonary pressure measured at end inspiration occlusion, *P*_*driv*_ Driving pressure, *P*_*L,driv*_ Lung driving pressure (i.e., difference between P_L,end-insp_ and P_L,end-exp_, *P*_*driv*_ driving pressure, measured by the difference between plateau pressure and total PEEP), *P*_*L,end-exp*_ Transpulmonary pressure measured at the end-expiratory occlusion, *MAP* Mean arterial pressure, *V*_*der*_ derecruited volume

Continuous variables were described as mean ± SD and compared using one-way analysis of variance with the Bonferroni post hoc test, or described as median [IQR] and compared using the Kruskal-Wallis test with Dunn’s post hoc test, as appropriate. Bonferroni adjustment was not used. (Refer to main text for explanations.) There is some variation in the number of measurements because of missing data. We report the number of measurements in detail for each variable in the additional files

^a^Variables reflecting the risks of overdistention and atelectasis were measured at clinical PEEP level

^b^ *P* < 0.05 PEEP-decreased vs. PEEP-unchanged

^c^Responses to an increment of PEEP in 3–5 cmH_2_O, expressed per 1-cmH_2_O PEEP increase

^d^Assessed by estimating the alveolar derecruitment with decreasing PEEP by 10 cmH_2_O

^e^ *P* < 0.05 PEEP-decreased vs. PEEP-increased

## Discussion

To our knowledge, this may be the first demonstration that systematic respiratory mechanics testing can be implemented with adequate validity and timelines in ICUs as a monitoring tool used for ventilatory adjustments. This provided physiological parameters for clinicians and helped to define the ventilatory therapy in patients with ARDS, as indicated by the changes in ventilator settings and in physiological variables observed after the test. Indeed, oxygenation could be improved using lower airway and distending pressures, a result that was unexpected, as indicated by the changes in OI, O/SI, Pplat, and ∆P. Such modifications would not have been performed without a systematic assessment.

### Validity of the measurements

Measurements of Pes in routine clinical practice out of the field of research have often been considered to be a challenge [2]. By implementing education sessions, standardized procedures, and an electronic form for automatic calculations, the measurements of Pes were conducted in our ICUs by a number of healthcare professionals (RTs, medical doctors) who were not experienced researchers. The ∆Pes/∆Paw ratio during the positive pressure occlusion test—a method used to validate appropriate positioning of the esophageal catheter and the Pes measurements—was consistent across our cohort and very close to unity, indicating that the obtained Pes was reliable and provided a valid measure of changes in pleural pressure. These ∆Pes/∆Paw ratios were also confirmed using occlusion tests against inspiratory effort [5] in six patients after resumption of spontaneous efforts.

### Changes in mode

The ventilator mode was often switched from pressure-targeted mode to volume-targeted mode, which may have been a direct effect of the test. An overall statistically significant but not clinically significant reduction in V_T_ was also observed after the measurements. Though there is no evidence showing that one mode is superior [13], VCV mode offers several advantageous features compared with PCV mode:

- Strictly controlling the V_T_ at a target value was proven beneficial for survival [14].
- Volume control permits an easier approach to monitoring respiratory mechanics. By setting an inspiratory pause time of 0.3–0.5 seconds, one can monitor Pplat in real time for each breath as well as its trend, leading to easy estimation of essential parameters such as Pdriv, compliance, and resistance. Peak Paw set in PCV is often used as a surrogate of Pplat, which potentially provides a simple method to limit Pdriv. This approach can overestimate Pplat when inspiratory flow does not return to zero at the end of inspiration; however, it can underestimate Pplat in the presence of the patient’s inspiratory effort.
- By using an inspiratory pause time with a high and constant inspiratory flow, VCV can improve the elimination of carbon dioxide [15].

### Changes in PEEP

**Fig. 3:**
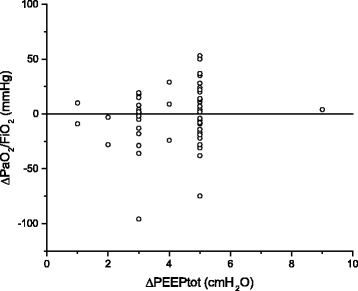
Individual oxygenation responses to the incremental positive end-expiratory pressure trial. *PaO*_*2*_ */FiO*_*2*_ *ratio* Ratio of partial pressure of arterial oxygen to fraction of inspired oxygen; *PEEPtot* Total positive end-expiratory pressure

A major change observed concerned the individualization of PEEP, as depicted in Fig. 2. Terragni et al. described how respiratory system mechanics may help in preventing lung-injury in patients with ARDS [12]. As shown in Table 3, the clinicians used the variables reflecting the risk of overdistention, the response to PEEP (see also the individual oxygenation response to PEEP in Fig. 3), and the recruitability to adjust PEEP, rather than the one reflecting the risk of atelectasis (P_L,end-exp_).

### Physiological effects

The changes in ventilator settings, especially a frequent reduction in PEEP level, eventually led to lower airway Pplat and Pdriv, and at the same time to improvements in oxygenation, OI, and O/SI (Table 2). This result was rather unexpected. We were surprised by the highly variable response to PEEP changes (Fig. 3). These results are different form the usual expectation of the effects of PEEP, but, in reality, they looked quite similar to recently published results of randomized clinical trials [16]. Oxygenation was improved but was not a primary physiological endpoint in our study for several reasons. With high Paw, improvements in oxygenation can be achieved by a reduction in cardiac output and shunt, which may worsen oxygenation delivery [17]. With high pressure, improvements in oxygenation have been associated with similar [18] or even worse mortality [14, 19]. OI takes into account both Paw and oxygenation, and it has been shown to be associated with survival as well as ∆P [8, 10, 20, 21]. We also found that the O/SI, as a new index to evaluate the benefit-to-cost ratio of mechanical ventilation, was significantly improved after the measurements. Although there are no data to support the possible association between O/SI and survival, we separated patients in our cohort by hospital outcome and found that O/SI (calculated using data from the measurements) in survivors was significantly higher than in nonsurvivors (14.6 [10.9-18.8] and 8.8 [6.0-12.6], respectively; *P* < 0.0005). We reasoned that the O/SI, similarly to OI, may be more meaningful than oxygenation itself regarding clinical outcome.

### Time points for comparisons

We decided to focus our comparison of the physiological variables at 1 h after the measurements to better ensure that the adjustments of ventilator settings and the physiological impact were likely secondary to the measurements. Later, the ventilation mode can be switched to partial assist mode. Also, comparing mean Paw (required for calculating OI) or Pdriv in pressure-target mode becomes challenging once the patient recovers spontaneous breathing effort.

We, however, also reviewed data at 24 h and 48 h. The improvements in physiological variables were consistent, indicated by the progressively lower OI at 24 and 48 h (12.4 ± 7.1 and 10.2 ± 0.9, respectively; *P* = 0.016) than at 1 h after the measurements. Of note, 16 and 21 patients (26.2% and 34.4%, respectively) had spontaneous effort, defined as actual RR exceeding preset RR or receiving partial support mode, – at 24 and 48 h.

### Limitations

Our study has limitations. First, though we tried to ensure that measurements were likely based on reasonable physiological principles, what we established was an association between the measurements and the changes in settings, owing to the nature of an observational study. Second, with no control group, we are not able to describe any results on outcomes. Third, the results are limited to a single center. Fourth, there were some missing data in the assessment, such as the response to PEEP and the recruitability. Fifth, the simplified method for estimating derecruitment at the bedside requires further validation. Sixth, although we tried to minimize a direct influence of the initiators of the project (LC, LB) on the ventilator settings, interactions were frequent at the beginning of the project, and it is difficult to ascertain their exact influence. These interactions were meant to overcome the technical and knowledge barriers to measurement and interpretation of respiratory mechanics in clinical practice.

## Conclusions

A respiratory mechanics test can be embedded in clinical practice and provides physiological parameters for clinicians. It leads to individualization of ventilator settings in patients with ARDS that improved physiological endpoints known to be associated with clinical outcomes, and it allowed reduction in the pressures needed to maintain oxygenation.

## Additional files

Additional file 1: Statistical analysis. **Figure S1** Patient flowchart. **Table S1** Spearman correlation between measurements and the clinical changes in PEEP*. **Table S2** Response standardized by ΔPEEP level and clinical adjustments of PEEP*. **Table S3** Arterial pH, bicarbonate, and vital signs before and after the measurements (*N* = 61)*. **Table S4** The detailed number of measurements for Table 3*. **Appendix S1** Sample of the custom-programmed PDF with data obtained in a real case. **Appendix S2 ** Sample of a clinical report automatically generated by the custom-programmed PDF. (DOCX 741 kb)

Additional file 2: Ethics approval letter. Ethic approval by the research ethics board (REB#16-095) of St. Michael’s Hospital (Toronto, ON, Canada). (PDF 307 kb)

## Abbreviations

ABG: Arterial blood gas
APACHE: Acute Physiology and Chronic Health Evaluation
ARDS: Acute respiratory distress syndrome
ECMO: Extracorporeal membrane oxygenation
FiO_2_: Fraction of inspired oxygen
ICU: Intensive care unit
IMV: Invasive mechanical ventilation
MAP: Mean arterial pressure
NIV: Noninvasive ventilation
OI: Oxygenation index
O/SI: Oxygenation/stretch index
PaCO_2_: Partial pressure of arterial carbon dioxide
PaO_2_/FiO_2_ ratio: Ratio of partial pressure of arterial oxygen to fraction of inspired oxygen
Paw: Airway pressure
PBW: Predicted body weight
PCV: Pressure-controlled ventilation
Pdriv: Driving pressure (Pplat − PEEPtot) or ∆P (Pplat − PEEP)
PEEP: Positive end-expiratory pressure
PEEPtot: Total positive end-expiratory pressure
Pes: Esophageal pressure
∆Pes/∆Paw: Ratio of changes in Pes and Paw during end-expiratory occlusion
P_L_: Transpulmonary pressure
P_L_: _end-exp_, Transpulmonary pressure at end expiration
P_L_: _end-insp_, Transpulmonary pressure at end inspiration
Ppeak: Airway peak pressure
Pplat: Airway plateau pressure
QI: Quality improvement
RR: Respiratory rate
RT: Respiratory therapist
SOFA: Sepsis-related Organ Failure Assessment
VCV: Volume-controlled ventilation
V_D_/V_T_,_esti_: Estimated physiological dead-space fraction
V_der_: Derecruited lung volume
V_E_,_corr_: Corrected expired volume per minute
V_E_: Minute ventilation
V_T_: Tidal volume
