# Supplementary material for: Implementing a bedside assessment of respiratory mechanics in patients with acute respiratory distress syndrome
Source: Crit Care. 2017 Apr 4;21:84. doi: 10.1186/s13054-017-1671-8 (PMC5379641; doi:10.1186/s13054-017-1671-8)
Supplement: Supplementary file 1 — Statistical analysis. Figure S1 Patient flowchart. Table S1 Spearman correlation between measurements and the clinical changes in PEEP*. Table S2 Response standardized by ΔPEEP level and clinical adjustments of PEEP*. Table S3 Arterial pH, bicarbonate, and vital signs before and after the measurements (N = 61)*. Table S4 The detailed number of measurements for Table 3*. Appendix S1 Sample of the custom-programmed PDF with data obtained in a real case. Appendix S2 Sample of a clinical report automatically generated by the custom-programmed PDF. (DOCX 741 kb) [file 13054_2017_1671_MOESM1_ESM.docx]

SUPPLEMENT OF THE MANUSCRIPT

Table of Contents

[*Statistical Analysis* 2](#_Toc477849615)

[Figure S1 Patient flowchart 3](#_Toc477849616)

[Table S1. Spearman correlation between measurements and the clinical changes in PEEP^*^ 4](#_Toc477849617)

[Table S2. Response standardized by delta PEEP level and clinical adjustments of PEEP* 5](#_Toc477849618)

[Table S3. Arterial pH, bicarbonate, and vital signs before and after the measurements (N=61)^*^ 6](#_Toc477849619)

[Table S4. The detailed number of measurements for Table 3* 7](#_Toc477849620)

[Appendix I: Sample of the custom-programmed PDF with data obtained in a real case 9](#_Toc477849621)

[Appendix II: Sample of a clinical report automatically generated by the custom-programmed PDF 11](#_Toc477849622)

# *Statistical Analysis*

Normality of the distribution was assessed by the Shapiro-Wilk test. Continuous variables are presented as means ± standard deviations or medians [interquartile ranges], as appropriate. Paired t-test was used to compare ventilator parameters and physiological variables before and after the measurements, if the within-subject differences had a normal distribution(2). A Wilcoxon matched pairs signed rank sum test was used in other cases. To analyze the relation between the physiological measurements and the subsequent clinical adjustments of PEEP, patients were divided into three groups according to their PEEP adjustment (post-measurement vs. pre-measurement: decreased, unchanged, and increased). We used a one-way analysis of variance (ANOVA) or a Kruskal-Wallis test to compare the respiratory mechanics between different PEEP adjustment groups, as appropriate. When analysis of variance indicated a significant difference, Bonferroni post hoc or Dunn’s test was used to identify differences between groups, as appropriate. We used a Spearman rank-order correlation analysis to investigate the association between the respiratory mechanics parameters and the adjustments in PEEP. A P value less than 0.05 was considered to indicate statistical significance. All P values are two-sided. All data were analyzed using Statistical Package for Social Sciences (SPSS, version 21.0). Because we decided the variables to be tested *a priori* in the statistical plan, we decided against using a Bonferroni correction which would highly increase the risk of type II errors (3).

# Figure S1 Patient flowchart


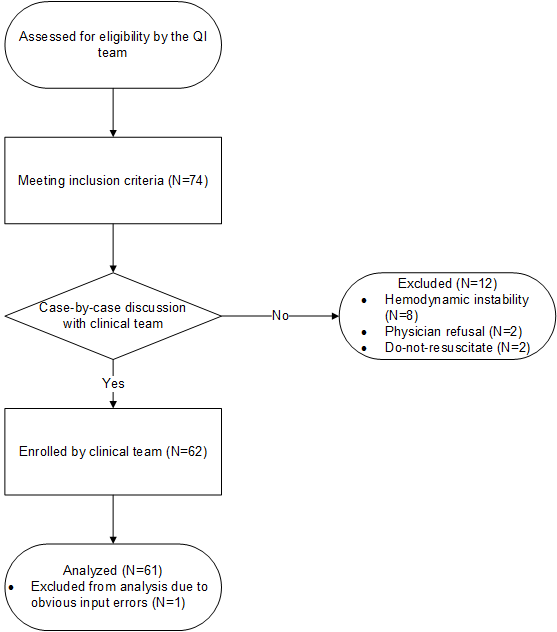


# Table S1. Spearman correlation between measurements and the clinical changes in PEEP^*^

| **Variables** | **N** | **Spearman’s rho** | **P value** |
| --- | --- | --- | --- |
| P_plat_ – cmH_2_O | 61 | -0.27 | 0.036 |
| Elastance-derived P_L, plat_ – cmH_2_O | 53 | -0.29 | 0.034 |
| Changes in P_L,driv_ – cmH_2_O | 47 | -0.30 | 0.042 |
| Vder – ml | 58 | -0.19 | 0.150 |

Abbreviations: *P_plat_ =* airway plateau pressure; *Elastance-derived P_L, plat_* = elastance-derived transpulmonary plateau pressure, calculated using airway plateau pressure times the ratio of lung elastance to respiratory system elastance; *P_L,driv_* = lung driving pressure, i.e. the difference between P_L,end-insp_ and P_L,end-exp_; *Vder = derecruited volume*

* Clinical changes in PEEP was calculated as the level at post-measurement minus the one at pre-measurement.

# Table S2. Response standardized by delta PEEP level and clinical adjustments of PEEP*

|  | **Clinical adjustments of PEEP (post-measurement vs. pre-measurement)** | | |  |
| --- | --- | --- | --- | --- |
|  | **Decreased (N)** | **Unchanged (N)** | **Increased (N)** | ***P* Value** |
| ***Response to the incremental PEEP trial*** ^§?^ | | | | |
| ∆P_driv_/∆PEEP | 0.57±0.72 (21) | 0.18±0.34 (27) | 0.04±0.07 (8) | **0.014** |
| ∆PL,_driv_/∆PEEP | 0.5±0.8 (17) | 0.1±0.4 (24) | -0.1±0.3 (6) | **0.047** |
| ∆PFratio/∆PEEP – mmHg/cmH_2_O | -0.9 [-5.6 to 3.7] (20) | -0.0 [-4.0 to 3.2] (26) | 2.2 [0.9 to 6.2] (8) | 0.256 |
| ∆MAP/∆PEEP – mmHg/cmH_2_O | -1.3±3.3 (18) | -0.5±1.2 (25) | -1.0±1.2 (7) | 0.537 |

Abbreviations: ∆P_driv_/∆PEEP = changes in driving pressure per an increment of PEEP; ∆PL,_driv_/∆PEEP = changes in lung driving pressure per an increment of PEEP; ∆PFratio/∆PEEP = changes in PaO2/FiO2 ratio per an increment of PEEP; ∆MAP/∆PEEP = changes in mean arterial pressure per an increment of PEEP.

Continuous variables were described as mean±SD and compared using the one-way ANOVA with Bonferroni post hoc test, or described as median [interquartile ranges] and compared using the Kruskal-Wallis test with Dunn’s test.

* We report changes in physiological variables per delta PEEP level because we changed PEEP by 3-5 cmH_2_O rather than by the same value between patients. Clinicians were able to raise PEEP for 3-5 cmH_2_O in most of our patients (N=46/56, 82%) to perform an incremental PEEP trial. A positive value means the change in physiological variable was in the same direction of change as PEEP, whereas a negative value means the opposite direction.

# Table S3. Arterial pH, bicarbonate, and vital signs before and after the measurements (N=61)^*^

| **Variables** | **Pre-measurement** | **Post-measurement** |
| --- | --- | --- |
| pH | 7.36 [7.29-7.40] | 7.37 [7.30-7.39] |
| Bicarbonate – mmol/L | 23[21-29] | 24[22-30] |
| Heart rate – bpm | 93[82-105] | 94[80-100] |
| Mean arterial pressure – mmHg | 79[72-88] | 79[70-90] |

* Continious variables were described as median [interquartile ranges].

# Table S4. The detailed number of measurements for Table 3*

|  | **PEEP (post-measurement vs. pre-measurement)** | | |  |
| --- | --- | --- | --- | --- |
| **Measured Variables** | **Decreased (N of measurements)** | **Unchanged (N of measurements)** | **Increased (N of measurements)** | ***P* Value** |
| ***Markers of overdistension*^†^** | | | | |
| **P_plat_ – cmH_2_O** | **28±5^#^ (23)** | **25±4^#^ (29)** | **26±2 (9)** | **0.013** |
| **Elastance-derived P_L, plat_ – cmH_2_O** | **21 [20-26]^#^ (19)** | **17 [16-20]^#^ (26)** | **18 [16-21] (8)** | **0.034** |
| **P_L, end-insp_ – cmH_2_O** | **5 [3-18] (19)** | **8 [4-9] (26)** | **4 [0-10] (8)** | **0.327** |
| **P_driv_ – cmH_2_O** | **15±5 (23)** | **12±4 (29)** | **13±3 (9)** | **0.098** |
| **P_L,driv_ – cmH_2_O** | **12±5 (19)** | **9±4 (26)** | **9±5 (8)** | **0.063** |
| ***Risk of atelectasis*^†^** | | | | |
| **P_L, end-exp_ – cmH_2_O** | **-2±5 (19)** | **-2±5 (26)** | **-5±5 (8)** | **0.335** |
| ***Response to the incremental PEEP trial* ^§^** | | | | |
| **Changes in P_driv_  – cmH_2_O** | **2.0 [0-3.5] (21)** | **1.0 [0-1.0] (27)** | **0.5 [0-12] (8)** | **0.169** |
| **Changes in P_L,driv_ – cmH_2_O** | **1.9±2.5 (17)** | **0.7±1.8 (24)** | **-0.3±1.2 (6)** | **0.042** |
| **Changes in PaO_2_/FiO_2_ – mmHg** | **-4 [-17 to 12] (20)** | **0 [-18 to 14] (26)** | **3 [-29 to 10] (8)** | **0.226** |
| **Changes in MAP – mmHg** | **-2 [-10 to 3] (18)** | **-2 [9 to 2] (25)** | **-3 [-8 to 0] (7)** | **0.675** |
| ***Recruitability*^‡^** |  |  |  |  |
| **Vder – ml** | **105±61^¶^ (22)** | **142±106 (28)** | **208±124^¶^ (8)** | **0.036** |

# Appendix I: Sample of the custom-programmed PDF with data obtained in a real case

# Appendix II: Sample of a clinical report automatically generated by the custom-programmed PDF

1. Ranieri VM, Rubenfeld GD, Thompson BT, Ferguson ND, Caldwell E, Fan E, Camporota L, Slutsky AS, Force ADT. Acute Respiratory Distress Syndrome The Berlin Definition. *JAMA-J Am Med Assoc* 2012; 307: 2526-2533.

2. Altman DG. Practical statistics for medical research. Boca Raton, Fla.: Chapman & Hall/CRC; 1999.

3. Perneger TV. What's wrong with Bonferroni adjustments. *BMJ* 1998; 316: 1236-1238.
