# Supplementary material for: Implementing a bedside assessment of respiratory mechanics in patients with acute respiratory distress syndrome
Source: Crit Care. 2017 Apr 4;21:84. doi: 10.1186/s13054-017-1671-8 (PMC5379641; doi:10.1186/s13054-017-1671-8)
Supplement: Supplementary file 2 — Ethics approval letter. Ethic approval by the research ethics board (REB#16-095) of St. Michael’s Hospital (Toronto, ON, Canada). (PDF 307 kb) [file 13054_2017_1671_MOESM2_ESM.pdf]

Research Ethics Office
Telephone: (416) 864-6060 Ext. 2557
Facsimile: (416) 864-6043
E-mail: pateld@smh.toronto.on.ca

St. Michael's

Inspired Care.
Inspiring Science.

May 11, 2016

Dr. Laurent Brochard
Department of Critical Care
St Michael's Hospital

Dear Dr. Brochard,

Re: REB# 16-095[C] - Impact of Systematic Assessment of Respiratory Mechanics in Patients With Acute Respiratory Distress Syndrome

**REB APPROVAL:**	**Original Approval Date**	**May 11, 2016**
	**Annual/Interval Review Date**	**May 11, 2017**

Thank you for your application submitted on **April 13, 2016**. The above noted study has been reviewed through a delegated process (not by Full Board review). The views of the St. Michael's Hospital (SMH) Research Ethics Board (REB) have been documented and resolved. Please note that no member of the St. Michael's Hospital Research Ethics Board associated with this study was involved in its review or approval.

The REB approves the study as it is found to comply with relevant research ethics guidelines, as well as the Ontario Personal Health Information Protection Act (PHIPA), 2004. The REB hereby issues approval for the above named study for a period of 12 months from the date of this letter. Continuation beyond that date will require further review of REB approval. In addition, the following documents have been reviewed and are hereby approved:

1. Protocol - ver: 4/8/2016

Furthermore, the following documents have been received and are acknowledged:

1. Master Linking Log - ver: 4/9/2016
2. Data Collection Form - ver: 1.0 4/9/2016

During the course of this investigation, any significant deviations from the approved protocol and/or unanticipated developments or significant adverse events should immediately be brought to the attention of the REB.

Please note that if a Clinical Trial Agreement is required, it must be submitted to the Office of Research Administration for review and approval. Any additional institutional approvals **must be** coordinated and approved through the Office of Research Administration prior to initiation of this research. All drug dispensing must be coordinated through the Research Pharmacy at 416-864-5413.

The St. Michael's Hospital (SMH) Research Ethics Board (REB) operates in compliance with the Tri-Council Policy Statement Ethical Conduct for Research Involving Humans, the Ontario Personal Health Information Protection Act, 2004, and ICH Good Clinical Practice Consolidated Guideline E6, Health Canada Part C Division 5 of the Food and Drug Regulations, Part 4 of the Natural Health Product Regulations, and the Medical Devices regulations. Furthermore, all investigational drug trials at SMH are conducted by Qualified Investigators (as defined in the latter document).

With best wishes

☐ Dr. David Mazer
Chair, Research Ethics Board

☒ Dr. Philip Berger
Vice Chair, Research Ethics Board

☐ Dr. Brenda McDowell
Vice Chair, Research Ethics Board

DM/PB/BJM/kt

St. Michael's Hospital, 30 Bond Street, Toronto, ON M5B 1W8 Canada T 416.360.4000
Fully affiliated with the University of Toronto. **stmichaelshospital.com**
